# T cell subpopulations in lymph nodes may not be predictive of patient outcome in colorectal cancer

**DOI:** 10.1186/1756-9966-30-78

**Published:** 2011-08-24

**Authors:** Roslyn A Kemp, Michael A Black, John McCall, Han-Seung Yoon, Vicky Phillips, Ahmad Anjomshoaa, Anthony E Reeve

**Affiliations:** 1 Cancer Genetics Laboratory, University of Otago, Dunedin, New Zealand; 2 Department of Biochemistry, University of Otago, Dunedin, New Zealand; 3 Department of Medical and Surgical Sciences, University of Otago, Dunedin, New Zealand; 4 Department of Pathology, University of Otago, Dunedin, New Zealand; 5 Department of Microbiology and Immunology, University of Otago, P.O. Box 56, Dunedin, New Zealand; 6 Human Genetics Division, Kerman University of Medical Sciences, Kerman, Iran

## Abstract

**Background:**

The immune response has been proposed to be an important factor in determining patient outcome in colorectal cancer (CRC). Previous studies have concentrated on characterizing T cell populations in the primary tumour where T cells with regulatory effect (Foxp3+ Tregs) have been identified as both enhancing and diminishing anti-tumour immune responses. No previous studies have characterized the T cell response in the regional lymph nodes in CRC.

**Methods:**

Immunohistochemistry was used to analyse CD4, CD8 or Foxp3+ T cell populations in the regional lymph nodes of patients with stage II CRC (n = 31), with (n = 13) or without (n = 18) cancer recurrence after 5 years of follow up, to determine if the priming environment for anti-tumour immunity was associated with clinical outcome.

**Results:**

The proportions of CD4, CD8 or Foxp3+ cells in the lymph nodes varied widely between and within patients, and there was no association between T cell populations and cancer recurrence or other clinicopathological characteristics.

**Conclusions:**

These data indicate that frequency of these T cell subsets in lymph nodes may not be a useful tool for predicting patient outcome.

## Background

Colorectal cancer is estimated to cause 639,000 deaths world wide per year [1]. The prognosis following surgery depends on disease stage, and this also determines the need for additional treatment. However clinico-pathological stage characteristics alone provide imperfect prognostic information. For example, approximately 25% of patients with disease localised to the primary site (UICC Stage I and II) relapse after surgery and may have benefited from adjuvant therapy [2], whereas 25% of patients with regional lymph node metastases (UICC Stage III) are cured by surgery alone [3]. Various ways to improve the prognostic accuracy of staging include increasing the number of lymph nodes analysed [4,5], increasing the sensitivity of the tests used to detect lymph node metastases [6] and using microarray technology to analyse gene expression [7,8]. However these methods do not take onto account potentially important host-related factors such as the immune response.

The immune response has long been associated with eradication of tumours [9]. More recently, it has become clear that T cells in the tumour are positively associated with good patient prognosis [10,11] in colorectal cancer. CD4 or CD8+ T cells expressing IFNγ, or the IFNγ inducing transcription factor Tbet, are the cells most likely involved at the tumour site [12,13].

In immune responses to infection, the effector CD4 and CD8 T cell populations are held in check by a third population of cells - regulatory T cells (Tregs). While there are numerous subtypes of T cells with regulatory function, the majority of suppressive function is mediated by Foxp3+ CD4+ Tregs. As expected, low numbers of these Foxp3+ Tregs have been associated with improved patient outcome in breast and colorectal cancers [14-16]. However, some authors report an association between *high *numbers of Tregs and positive patient outcome [17,18], although Salama et al found a negative association between patient outcome and high frequency of Tregs in the non-tumour associated tissue [18]. More recently, Chaput et al identified a population of CD8+Foxp3+ T cells in a cohort of colorectal cancer patients that had suppressive activity and were proposed to mediate tumour escape [19].

The immune response is initiated in the lymph nodes, and although analyses of T cell subsets in the lymph nodes of breast cancer patients have been performed [20], the effect of these T cell subsets on colorectal cancer patient outcome had not been explored. We hypothesised that the priming environment of an anti-tumour immune response would be a useful predictor of patient outcome. In this study we examined the lymph nodes of Stage II colorectal cancer patients to identify CD4+, CD8+ and Foxp3+ cell populations and correlated these with patient outcome, alone, and in combination with other clinico-pathological variables.

## Methods

### Patients

Patients with UICC stage II colon cancer were included in this study. Stage II patients were chosen because they have no tumour metastases in lymph nodes. The number of lymph nodes retrieved from patients for staging is indicated in Table 1. Approximately 50% of the lymph nodes obtained from each patient were randomly selected for immunohistochemical analysis.

**Table 1:** Clinical characteristics of patients

		*CRC - recurrent*	*CRC - non recurrent*	*IBD controls*
Number patients		13	18	9
Age (years, mean (SD))		70.84 (8.922)	72.24 (11.032)	
Gender %				
M		39	28	
F		61	72	
				
Differentiation	Poor	1	3	
	Moderate	11	14	
	Well	1	1	
Tumour Site	Right	8	13	
	Left	5	2	
	Rectum	0	1	
Number lymph nodes used for staging (mean (SD))		20 (12)	19 (8)	
Number lymph nodes analysed (mean (SD))		10 (6)	11 (8)	5 (3)

All patients underwent elective surgery for colon cancer at Dunedin Hospital, New Zealand. Pathological staging was verified by the study pathologist (HSY). In addition to colon cancer, patients with inflammatory bowel disease were used as controls. The study was approved by the Lower South Regional Ethics Committee and patients gave signed informed consent to participate. All patients were prospectively followed up for a minimum of five years from the date of surgery.

### Immunohistochemical Analysis

Formalin fixed paraffin embedded (FFPE) lymph nodes recovered at surgery were used for immunostaining. 4 um serial sections were stained for T cell markers using two methods. Tonsil tissues were used as positive and negative controls.

### CD4 and CD8

Sections were dried for 30 min after cutting, then dewaxed on the Bond™ (Leica Microsystems, Germany) after manual drying. Heat induced epitope retrieval was performed using ER2 (Bond™) at pH 9.0 for 20 min at 100°C. After blocking with 3% peroxide block for 5 min, the sections were incubated with the specific antibody (anti-human CD4 (NCL-L-CD4-368; Novocastra, Leico Microsystems; 1:40 dilution) or anti-human CD8 (NCL-CD8-4B11; Novocastra, Leico Microsystems; 1:100 dilution)) for 20 min at RT. Unbound antibody was removed by 3 washes in Bond™ Wash Solution before adding polymer for 10 min at RT. After washing unbound labeled polymer in Bond™ Wash Solution 3 times, peroxidase staining in tissue sections was revealed by DAB solution (Bond™). After stopping the reaction in running water, sections were counter-stained with a rinse in hematoxylin solution. After dehydration, the sections were mounted with DPX.

### Foxp3

According to published methods [21], slides were incubated with rat anti-human Foxp3 antibody (clone PCH101, dilution 1:200, eBioscience, San Diego, CA) for 1 h at room temperature, followed by goat anti-rat antibody (dilution 1:50, Zymed) and ABC peroxidase detection system (Vector Vectastain ABC Elite kit, Vector Laboratories, Burlingame, CA).

Between 1 and 33 lymph nodes per patient (Table 1) were analysed with a Zeiss microscope (Carl Zeiss Co., Oberkochen, Germany) in their entirety to eliminate regional variation due to the complex architecture of lymph nodes. Each field was recorded using SpotOn software (Brookvale, Australia) and CD4, CD8 and Foxp3+ cells quantified using Image J software (NIH, USA). Frequency of positively stained cells compared with total cells was acquired for each field. All samples were analysed in a double-blinded fashion.

### Statistical analysis

Frequency counts of CD4, CD8 and Foxp3 stained cells from each field were logged to reduce data skewness, with an offset used to adjust zero counts. For each T-cell marker the R statistical software [22] was used to fit a linear mixed model to the logged count data, with a fixed effect term used to represent clinical variables, and random effects for patient number and lymph node. A separate model was used for each of the available clinical variables: (disease status, differentiation, lymphatic invasion, margin, tumour site). In each model linear contrasts were used to assess the presence of differences in logged counts between each of the three disease status groups for each T-cell marker. An identical approach was taken in the analysis of log-ratio data for pairs of T-cell markers (CD4:Foxp3, CD8:Foxp3), with the log-ratios of counts derived using matched fields from within each lymph node.

## Results

Thirty three patients with stage II colon cancer were included; 13 with and 18 without recurrence after 5 years of follow up. Of the 13 patients with recurrent disease, four recurred locally and nine had systemic disease (seven liver, one lung, and one lung and brain). Patient characteristics are summarised in Table 1. For each patient, between 1 and 33 lymph nodes were available for analysis (median = 10). Within each lymph node, between one and 15 sections were examined for CD4, CD8 and FoxP3 percentage (median = 10). For those nodes for which multiple sections were available, the "within-node" standard deviation was calculated to assess the consistency of immunological signal being obtained. Similarly, for those patients from whom multiple lymph nodes were sampled, the "within-patient" (i.e., "between-node" for the same patient) standard deviation was calculated. Finally the average immunological "signal " was calculated for each patient (for each of FoxP3, CD8 and CD4) and used to assess inter-patient variability by determining the "between patient" standard deviation.

**Figure 1:**
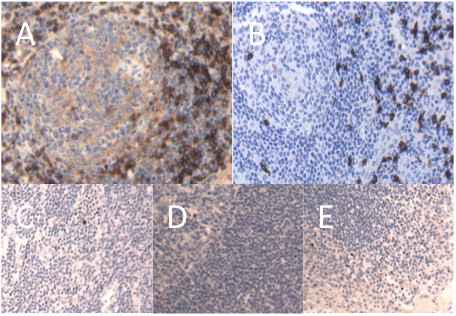
**Sections from representative regional lymph nodes showing positive staining for CD4, CD8 or Foxp3**. Lymph node sections were stained for CD4 (A), CD8 (B) or Foxp3 (C) as outlined in Materials and Methods. Foxp3 staining was optimised using tonsil tissue - negative (D) and positive (E) control samples are shown. Representative samples are shown.

Figure 1 shows immunohistochemical staining for CD4, CD8 and Foxp3 respectively. For all three measures of immunological activity (CD4, CD8 and FoxP3), the within-node variability was around half the level of the within-patient (between-node) variability (CD4: 5.81% vs 10.40%, CD8: 2.25% vs 4.24%, FoxP3: 0.24% vs 0.63%), indicating that replicate measurements obtained from the same node were relatively consistent in all cases. The same was not true, however, of nodes taken from the same patient, with the between-node standard deviation approximately the same as the between-patient standard deviation for all three measures of immunological activity (CD4: 10.40% vs 9.12%, CD8: 4.24% vs 4.15%, FoxP3: 0.63% vs 0.68%). That is, the variation in CD4, CD8 and FoxP3 percentages between nodes from the same patient was as great as the variation observed from one patient to another.

Given the large amount of within-patient variability that was observed across multiple lymph nodes from the same patient, the task of identifying differences in immunological activity between different groups of patients could be expected to be very challenging, as is reflected in the results presented below.

### No association between T cell frequency in the lymph nodes and patient outcome

**Figure 2:**
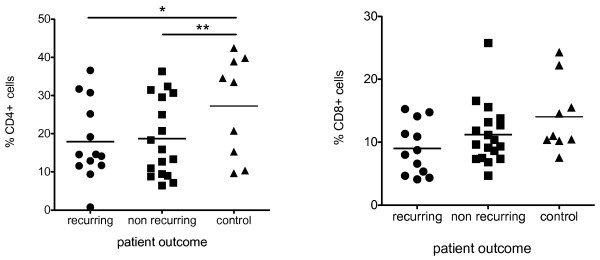
**No association between CD4+ or CD8+ cells and patient outcome**. Between 1 and 20 lymph nodes per patient (Table 1) were analysed for CD4 or CD8+ cells as indicated. Control lymph nodes came from patients diagnosed with inflammatory bowel disease. Data are represented as mean +/- SEM. * P = 0.095, ** p = .0669.

There was no association between the frequency of either CD4+ or CD8+ cells and cancer recurrence (Figure 2). There was a difference in the frequency of CD4 cells in the inflammatory bowel disease control cohort (mesenteric lymph nodes from healthy controls were unavailable). This was not unexpected given that these patients have a chronic inflammatory disease that involves CD4 T cells [23].

### No association between Foxp3+ cells in the lymph nodes and patient outcome

**Figure 3:**
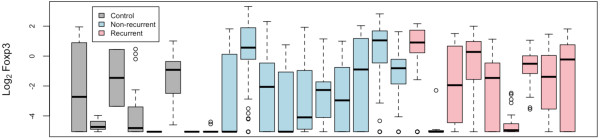
**No association between Foxp3+ cells and patient outcome**. Between 1 and 20 lymph nodes per patient (Table 1) were analysed for Foxp3+ cells. Control lymph nodes came from patients diagnosed with inflammatory bowel disease. Data are represented as logged (base two) cell counts, with each boxplot representing the distribution of mean log_2 _Foxp3 cell counts for each lymph node of a single patient.

Although there was no difference in the percentage of T cells between patients with and without cancer recurrence, it was possible a subpopulation of cells was associated with disease. Because Tregs are important in tumour immune responses, we analysed the frequency of this cell population in the lymph nodes. Both CD4 and CD8 Tregs can express Foxp3 [15,19], and so we used this marker to measure the frequency of Tregs in a subset of patients from each group (control, recurrent and non-recurrent) in Figure 2; these patients were selected on availability of lymph node samples. No association was found between frequency of CD4+Foxp3+ or CD8+Foxp3+ cells and cancer patient outcome (Figure 3). Furthermore, no association was found between frequency of CD4+Foxp3+ or CD8+Foxp3+ cells in cancer patients and control IBD patients. This last finding was interesting considering previous work that suggests Tregs are decreased in IBD patients compared to healthy controls [24]. It is possible that the cancer patients are also presenting with an inflammatory phenotype, but we were unable to make a comparison with lymph nodes from healthy control subjects.

### Association between T cell populations and other clinico-pathological variables

**Figure 4:**
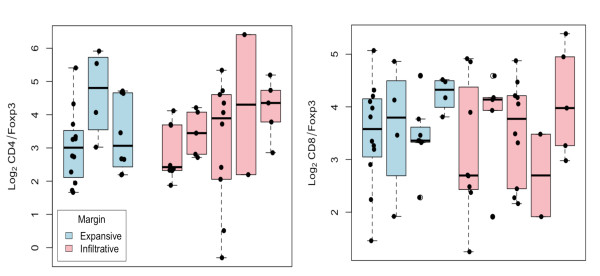
**No association between Foxp3+ cells as a subset of CD4 T cells and tumour clinical features**. Between 1 and 20 lymph nodes per selected patients with data available regarding tumour margin were analysed for Foxp3+ cells as a ratio of CD4+ (A) or CD8+ (B) cells. Data are represented as logged (base two) cell count ratios, with each boxplot representing the distribution of mean log_2 _ratios for each lymph node of a single patient. Solid circles indicate actual log-ratio values.

The relationship between CD4, CD8 or Foxp3 positive cells with clinico-pathological variables was examined (differentiation, lymphatic invasion, tumour margin, tumour site, vascular invasion). No significant associations between T cell subsets and these other variables were identified (data not shown). However, it seemed possible that the frequency of Foxp3 cells as a subset of CD4+ or CD8+ cells could correlate with clinical parameters. Analysis of this ratio and tumour margin showed no association (Figure 4).

## Discussion

In this paper, we have described the analysis of T cell populations in the lymph nodes of Stage II colorectal cancer patients. We were unable to find any association between CD4, CD8 or Foxp3+ (presumed Tregs) and cancer recurrence or with other clinico-pathological variables.

T cells have long been known to play a role in eradicating tumours. Colorectal cancer has been particularly well studied, with several laboratories showing a positive association between patient survival and effector (IFNγ+) T cell infiltration into the tumour [10,11]. It was expected that the regulatory T cell infiltration into the tumour would be negatively associated with patient outcome; however, regulatory (FoxP3+) T cells have been shown to have a protective role in colorectal cancer, in contrast to their negative role in many other cancers [17]. The positive effect of FoxP3+ T cells has been proposed to be a result of their effects on other T cells that are promoting tumour growth [25].

T cell immune responses are initiated in the lymph nodes by cells, such as dendritic cells, presenting tumour antigens to responding specific T cells. These activated T cells then migrate to the tumour and specifically destroy it. Munn et al proposed that the tumour draining lymph node is a unique immunological environment where the presence of regulatory T cells could mediate a suppressive effect on anti-tumour immune responses [26]. Indeed, depletion of Tregs enhances effector T cell responses in tumour draining lymph nodes [27]. Recent data also indicated that the presence of Foxp3+ T cells in tumour draining lymph nodes of colorectal cancer patients correlated with disease progression [28]. Given the associations between Treg infiltration in primary colorectal tumours and patient outcome [18], we questioned whether Tregs in the regional lymph nodes could be predictive of patient survival.

Our data is in contrast to Khort et al [20], who described a population of CD4 cells in the axillary lymph node could predict outcome in breast cancer patients. Although our sample was smaller, there were no apparent trends in the data to indicate that a larger sample would be likely to yield significant results. In fact, given the amount of variation in immunological activity that we observed in lymph nodes taken from the same patient, the use of lymph nodes for prognostic purposes would seem to be extremely challenging. Even if a difference in activation existed between patients with "good" and "poor" prognosis, detection of a statistically significant difference would require collection of large numbers of both patients and nodes. For per-patient prognosis, the inter-node variability would make accurate prediction almost impossible, with the good and poor responders likely to be indistinguishable from one another. This is likely due to the background of non tumour-specific T cell overshadowing the presence of tumour specific responses - indeed, the majority of studies looking at T cells as predictors of outcome in this disease have been restricted to the tumour tissue [11,12,17,18,21,29].

We did not identify the sentinel nodes, which are believed to be the primary priming site for the anti-tumour immune response, however data exists to indicate that there is often more than one sentinel node and it's spatial relationship to the tumour can vary considerably [30].

Immunotherapy of cancer patients is difficult due to the specific nature of the adaptive immune response and the absence of easily identifiable tumour specific antigens. The current study looked only at total T cell populations in the lymph node, and it may be that tumour specific T cell populations were present in different frequencies in patients with and without recurrence, but not able to be identified as such.

A further complication is the lack of healthy control tissue. Studies comparing immune response in colorectal cancer patients have used blood of healthy patients [14,15]; however the scope of our study was to investigate the role of lymph nodes for predicting patient outcome, and mesenteric lymph nodes from healthy controls were not obtainable. We compromised by using matching lymph node tissue from IBD patients, as has been previously published [15] but are aware of the difficulties of using immune tissue from patients with an immune mediated inflammatory disease.

However, an interesting finding was the difference between colorectal cancer patients and inflammatory bowel disease patients with respect to CD4 expression. IBD patients had a higher CD4 frequency that is not surprising given the inflammatory nature of IBD and the proven role for CD4 cells in driving this disease [23]. However, no difference was seen between cancer patients and IBD patients in Foxp3+ cells. This indicates that the Treg population was not diminished in IBD patients, a finding in direct contrast to Clarke et al. We are currently investigating this further to examine the role of other T cell subpopulations.

Foxp3 is recognised as the most specific Treg marker; however, there are reports of Foxp3 expression in effector T cells, especially in humans [31]. It is possible that the Foxp3 cells detected in our study were effector rather than regulatory cells. Studies are underway to further characterise these cells, using a panel of regulatory markers. Clarke et al found that Foxp3+ cells recovered from mesenteric lymph nodes of CRC patients exhibited regulatory activity against CD4 T cells [15], so it seems likely that Foxp3+ cells in our study have regulatory function.

## Conclusions

We found no correlation between major T cell populations in regional lymph nodes and cancer recurrence in patients with stage II colon cancer. A more detailed analysis of T cell sub-populations will be required to determine whether characterisation of the immune response in regional lymph nodes can inform prognosis in colorectal cancer.

## Competing interests

The authors report no conflicts of interest with people or organizations that could inappropriately influence the work. The authors did not receive any outside assistance writing this manuscript.

## Authors' contributions

RAK conceived of the study, designed and performed experiments, and drafted the manuscript. MAB performed all statistical analyses and helped draft the manuscript. JM coordinated clinical samples and helped draft the manuscript. HSY, VP and AA participated in experimental design and interpretation. AER coordinated the study. All authors read and approved the final manuscript.
